# PGC-1α Is a Key Regulator of Glucose-Induced Proliferation and Migration in Vascular Smooth Muscle Cells

**DOI:** 10.1371/journal.pone.0004182

**Published:** 2009-01-14

**Authors:** Lingyun Zhu, Guoxun Sun, Hongjie Zhang, Yan Zhang, Xi Chen, Xiaohong Jiang, Xueyuan Jiang, Stefan Krauss, Junfeng Zhang, Yang Xiang, Chen-Yu Zhang

**Affiliations:** 1 Jiangsu Diabetes Center, State Key Laboratory of Pharmaceutical Biotechnology, School of Life Sciences, Nanjing University, Nanjing, People's Republic of China; 2 Department of Gastroenterology, the First Affiliated Hospital of Nanjing Medical University, Nanjing, People's Republic of China; 3 Department of Cancer Biology and Therapeutics, Merck Research Laboratories, Boston, Massachusetts, United States of America; University of Cambridge, United Kingdom

## Abstract

**Background:**

Atherosclerosis is a complex pathological condition caused by a number of mechanisms including the accelerated proliferation of vascular smooth muscle cells (VSMCs). Diabetes is likely to be an important risk factor for atherosclerosis, as hyperglycemia induces vascular smooth muscle cell (VSMC) proliferation and migration and may thus contribute to the formation of atherosclerotic lesions. This study was performed to investigate whether PGC-1α, a PPARγ coactivator and metabolic master regulator, plays a role in regulating VSMC proliferation and migration induced by high glucose.

**Methodology/Principal Findings:**

PGC-1α mRNA levels are decreased in blood vessel media of STZ-treated diabetic rats. In cultured rat VSMCs, high glucose dose-dependently inhibits PGC-1α mRNA expression. Overexpression of PGC-1α either by infection with adenovirus, or by stimulation with palmitic acid, significantly reduces high glucose-induced VSMC proliferation and migration. In contrast, suppression of PGC-1α by siRNA mimics the effects of glucose on VSMCs. Finally, mechanistic studies suggest that PGC-1α-mediated inhibition of VSMC proliferation and migration is regulated through preventing ERK1/2 phosphorylation.

**Conclusions/Significance:**

These results indicate that PGC-1α is a key regulator of high glucose-induced proliferation and migration in VSMCs, and suggest that elevation of PGC-1α in VSMC could be a useful strategy in preventing the development of diabetic atherosclerosis.

## Introduction

Atherosclerosis and microvascular diseases are the major vascular complications of diabetes, and constitute the principal causes of morbidity and mortality among diabetics [1], [2]. While many factors potentially contribute to the development of diabetic atherosclerosis including abnormalities in plasma lipoproteins and blood pressure, hyperglycemia is generally believed to be a major causative factor [3]. Chronic hyperglycemia-induced atherosclerosis involves a complex series of events, including abnormal vascular smooth muscle cell (VSMC) proliferation and migration, which contribute importantly to the formation of organized atherosclerotic plaque. High glucose activates the expression of several genes involved in extracellular signal-regulated kinase (ERK)-dependent mitogenic response, contributing to VSMC proliferation and migration and, as a result, to the development of atherosclerosis [4]–[6].

Recent studies demonstrated that TZDs, the PPARγ ligands, decreased cardiovascular risks via exerting direct effects on vascular cells, for example, inhibition of VSMC proliferation and migration [7], [8]. It has been shown that TZDs inhibit key steps in the ERK/MAPK pathway, blocking events that are critical for the re-entry of quiescent VSMCs into cell cycle, thus retarding serum-induced growth of cultured arterial VSMCs and PDGF-BB–directed migration of VSMCs [9]–[12]. Direct vascular effects of TZDs result from their activity as ligands for the nuclear receptor, PPARγ [7].

PGC-1α is a transcriptional coactivator of PPARγ and regulates expression of many genes coding for mitochondrial proteins [13]–[15]. PGC-1α plays important roles in many physiological processes, including adipocyte cell differentiation [13], the regulation of adaptive thermogenesis in brown fat and muscle [13], [16], control of hepatic gluconeogenesis [17], fuel selection in skeletal muscle cells [18], and suppression of reactive oxygen species and neurodegeneration [19]. Previous data indicate that PGC-1α expression changes in tissues depending on glucose levels, as seen in diabetic subjects [17], [20]–[23], thus mediating effects of hyperglycemia and promoting pathological conditions. For example, PGC-1α is upregulated in liver of rodents in models of both type 1 and type 2 diabetes, and increased PGC-1α expression contributes to elevated hepatic glucose output and the development of hyperglycemia [17]. Here, we sought to establish whether hyperglycemia regulates PGC-1α levels in VSMCs and whether such changes are causally related to hyperglycemia-induced VSMC proliferation and migration. Our results suggest that PGC-1α is a glucose-responsive, negative regulator of VSMC proliferation and migration.

## Results

### High Glucose-induced VSMC proliferation and migration is associated with decreased PGC-1α expression

**Figure 1:**
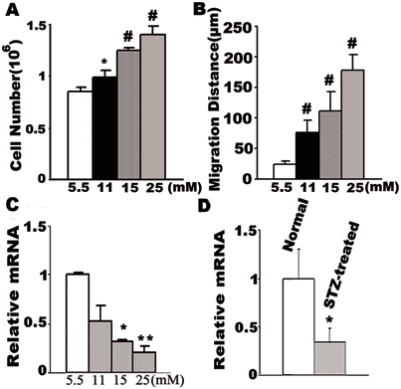
High Glucose-induced VSMC proliferation and migration is associated with decreased PGC-1α expression. Cultured rat VSMCs were incubated for 48 h in 5.5 mM, 11 mM, 15 mM and 25 mM glucose respectively, Cell growth was determined by counting the number of cells (A) and VSMC migration was determined by a standard wound healing assay (B), PGC-1α expression was determined by real-time RT-PCR by using primers specific for rat PGC-1α and β-actin (C). The bars represent means±S.E.M (n = 6). *P<0.05, **P<0.01, #P<0.001 vs. cells incubated in 5.5 mM glucose. Arterial samples were obtained from the normal and STZ injected rats, the intima and outer and inner tissue layers were removed from arteries. PGC-1α expression was determined by real-time RT-PCR. Data (n = 10) were expressed as means±S.E.M. *P<0.05 vs. normal rats (D).

High glucose has been reported to increase rat VSMC growth and movement [3], [6]. To confirm the previous report, cultured rat VSMCs were incubated with various concentrations of glucose. Following 48 h incubation, glucose dose-dependently induced VSMC proliferation (Fig. 1A) and migration (Fig. 1B). 11 mmol/L glucose induce VSMC proliferation by 1.2-fold and VSMC migration by 3-fold, respectively. At 15 mmol/L glucose numbers of VSMC were nearly 1.5-fold higher than that of control group, while migration distance was increased by 4-fold compared to the control group. 25 mmol/L glucose further stimulated cell growth by 1.6-fold and cell migration by 6-fold.

The glucose-induced VSMC proliferation and migration was found to be associated with the inhibition of the expression of PGC-1α. As shown in Fig. 1C, 11 mmol/L glucose inhibited PGC-1α mRNA expression in rat VSMCs to 52±24% relative to the 5.5 mmol/L control, at 15 mmol/L glucose PGC-1α mRNA expression was 31±2% of control, and 25 mmol/L glucose (the highest glucose condition) reduced the PGC-1α mRNA expression to 20±7% of control.

We also examined PGC-1α mRNA expression in the blood vessel media (VSMC is the only cell type in this layer) in normal and STZ injected rat arterial samples. In STZ-treated rats, PGC-1α mRNA level was decreased by 66±14% compared to the control group (Fig. 1D).

### Overexpression of PGC-1α suppresses high glucose induced VSMC proliferation and migration

**Figure 2:**
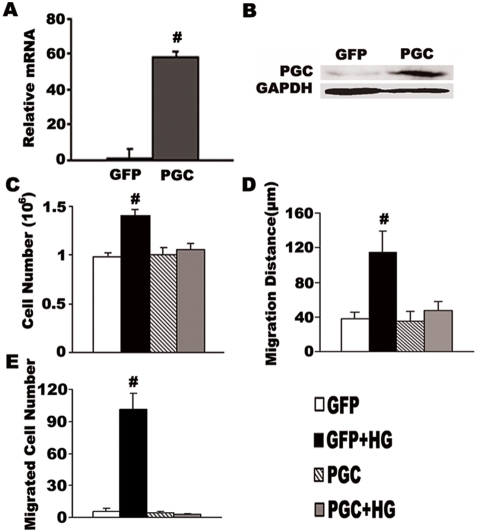
Overexpression of PGC-1α suppresses high glucose induced VSMC proliferation and migration. Cultured rat VSMCs in 5.5 mM glucose infected with adenovirus driving the expression of PGC-1α (PGC group) or GFP (GFP group) were either left untreated or incubated with 15 mM gluoose (HG) for 48 h. PGC-1α mRNA expression was determined by real-time RT-PCR (A), the values of PGC-1α/β-actin were normalized to that of control. 60 µg total proteins from VSMCs were assayed for Westernblot with PGC-1 antibody (B). Cell growth was determined by counting the number of cells (C). VSMC migration was determined by a standard wound healing assay (D) and by transwell analysis (E). The bars represent means±S.E.M (n = 6). **P<0.01, #P<0.001 compared with the GFP control group (GFP group).

To further study the role of PGC-1α in VSMC proliferation and migration, VSMCs were infected with adenovirus driving the overexpression of PGC-1α. After 24 h PGC-1α mRNA and protein level was markedly elevated. As shown in Fig. 2A and 2B, after infection with Ad-PGC-1α, the PGC-1α mRNA level in VSMCs was approximately 60-fold higher than negative control group (Ad-GFP) (Fig. 2A), and PGC-1α protein level also increased significantly (Fig. 2B). Subsequently added high glucose did not affect this elevation (data not shown).

15 mmol/L glucose was used to stimulate VSMC growth and movement, the number of Ad-GFP infected cells, cultured in 15 mmol/L glucose were nearly 1.5-fold higher than cells cultured at 5.5 mmol/L glucose (1.41±0.07×10^6^ cell/dish versus 0.98±0.03×10^6^, P<0.001, n = 6, Fig. 2C). High glucose also significantly stimulated cell migration in Ad-GFP infected group, as determined by wound healing assay (37.33±8 versus 114.67±25.33 µm, P<0.001, n = 6, Fig. 2D) and transwell chamber assay (6±2.9 versus 101±15.7; P<0.001, Fig. 2E). Strikingly, when cells were infected with Ad-PGC-1α, the ability of high glucose to induce VSMC proliferation was entirely lost (1.01±0.06×10^6^ versus 1.05±0.06×10^6^ cells/dish, P>0.05, n = 6, Fig. 2C). The wound healing assay showed that high glucose-induced VSMC migration was significantly decreased after overexpression of PGC-1α compared to control cells cultured at high glucose (35.55±11.10 versus 114.67±25.33 µm, P<0.001, n = 6, Fig. 2D). A similar result was seen in the transwell chamber assay, where the number of migrated cells in high glucose was 4±1 following overexpression of PGC-1α compared to 101±15.7 in the Ad-GFP infected cells (also at 15 mmol/L glucose) (Fig. 2E). Note that, under high glucose conditions, cells overexpressing PGC-1α essentially showed the same migration distance and number of migrated cells as the control cells (GFP group without high glucose stimulation). In summary, using multiple assay formats, overexpression of PGC-1α clearly suppresses high glucose-induced VSMC proliferation and migration.

### Knock-down of PGC-1α by siRNA accelerates high glucose-induced VSMC proliferation and migration

**Figure 3:**
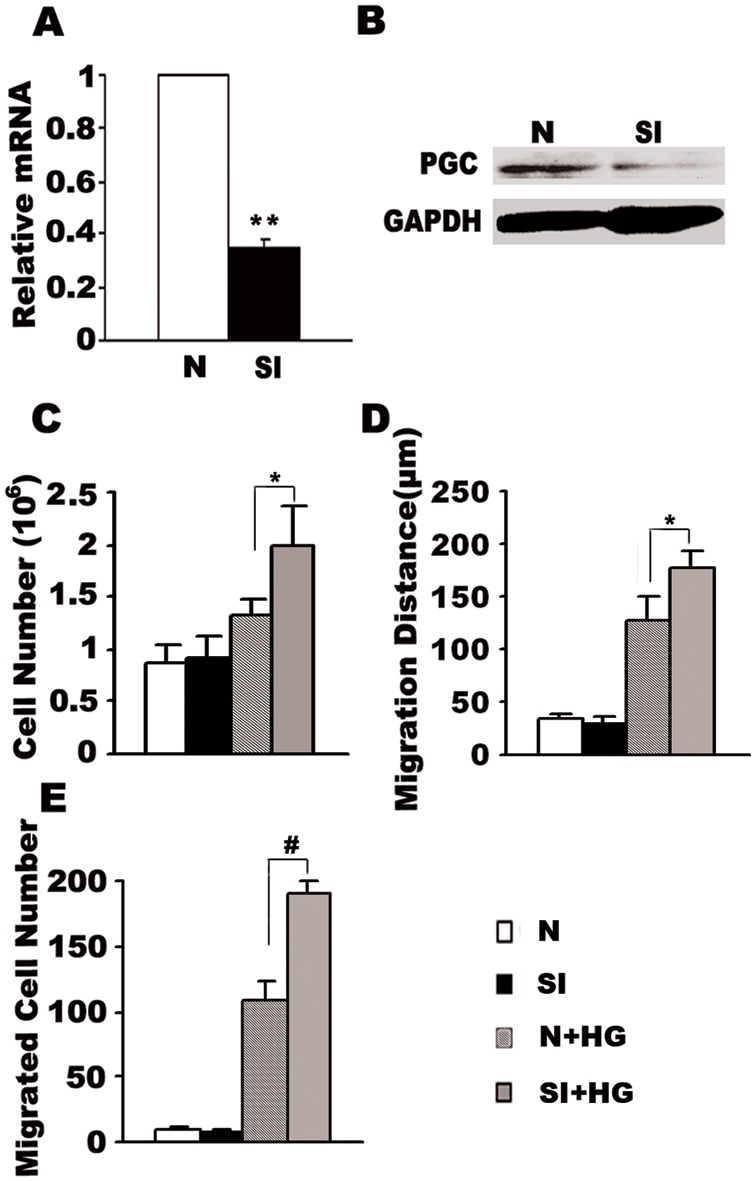
Knock-down of PGC-1α accelerated high glucose-induced VSMC proliferation and migration. VSMCs transfected with siRNA (SI) or the negative control (N), were either left untreated or stimulated with 15 mM gluoose (HG). The interference effect was assessed by quantitative PCR analysis and data were shown as the ratio of PGC-1α/β-actin (A), **P<0.01compared with the negative control group (N). 120 µg total proteins from VSMCs were assayed for Western blot with PGC-1 antibody to confirm the interference effiency (B). Effects of decreased PGC-1α on high glucose-induced VSMC proliferation were determined by cell counting (C). VSMC migration was determined by wound healing (D) and transwell analysis (E). Individual data points in this figure represents the mean±S.E.M (n = 6). *P<0.05, #P<0.001 compared with the control group (N+HG).

To further test the effect of PGC-1α on VSMC proliferation and migration, we knocked down PGC-1α expression in VSMCs by siRNA interference. As shown in Fig. 3A and 3B, our siRNA inhibited PGC-1α expression to about 30%. In contrast to the inhibitory effect of elevated PGC-1α on VSMC growth, decreased PGC-1α level accelerated high glucose-induced VSMC proliferation and migration. Under high glucose conditions (15 mmol/L as in the previous experiments), knocking down PGC-1α increased VSMC number compared to control (1.99±0.37×10^6^ versus 1.32±0.15×10^6^ cells/dish, P<0.05, n = 6, Fig. 3C). In addition, following PGC-1α knock-down, VSMC migration distance was further induced under high glucose conditions when compared to the negative control group (N) (176.25±17.81 µm versus 126.25±23.82, P<0.05, n = 6, Fig. 3D). Finally, transwell analysis confirmed that PGC-1α knock-down increased the number of migrated VSMCs compared to control (190.20±9.78 versus 109.4±14.40, P<0.001, Fig. 3E).

### Palmitic acid stimulates PGC-1α expression and inhibits high glucose-induced VSMC growth and movement

**Figure 4:**
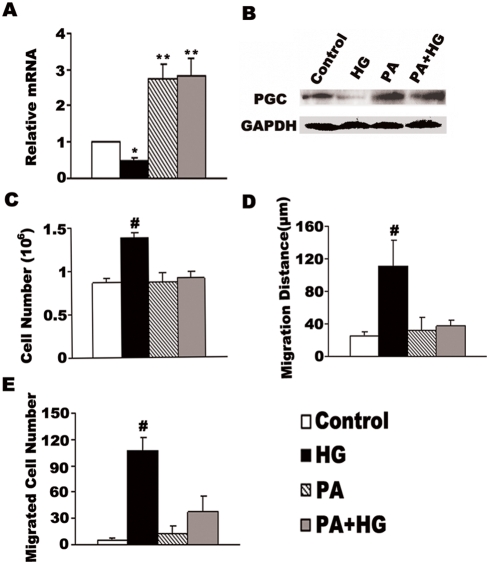
Palmitic acid stimulates PGC-1α expression and retards high glucose-induced VSMC growth and movement. Cultured rat VSMCs in 5.5 mM glucose were either left untreated or stimulated with 15 mM glucose (HG) in the absence and presence of 0.4 mM palmitic acid (PA) for 48 h. PGC-1α expression was determined by real-time RT-PCR (A) and by Western blot with PGC-1 antibody compared with GAPDH antibody, 120 µg total proteins from VSMCs were loaded (B). Cell growth was determined by counting the number of cells (C). Migration distance was determined by a standard wound healing assay (D) and transwell analysis (E). The bars represent means±S.E.M (n = 6). **P<0.01 compared with control conditions, #P<0.001 compared with the control group.

In a previous study, we had shown that palmitic acid stimulates endogenous PGC-1α expression in VSMCs [24]. Therefore, we used palmitic acid in these experiments as yet another way of modulating PGC-1α and assessing the effects on cell growth and proliferation. 0.4 mmol/L palmitic acid alone increased PGC-1α mRNA level 2.7-fold after 48 h incubation, the protein level also increased after treatment with 0.4 mmol/L palmitic acid (Fig. 4A and 4B). The effect of palmitic acid on PGC-1α expression was not reversible by glucose, as shown by concomitant incubation of VSMCs with 15 mmol/L glucose and 0.4 mmol/L palmitic acid (Fig. 4A and 4B).

Relative to control, palmitic acid alone had no effect on VSMC proliferation (Fig. 4C) and migration(Fig. 4D and 4E), however VSMC proliferation and migration were kept to the level of the control when cells were incubated with high glucose plus palmitic acid. As shown in Fig. 4C, incubation of VSMCs with 0.4 mmol/L palmitic acid in the presence of 15 mmol/L glucose ablated the effect of high glucose on cell growth. (0.92±0.07×10^6^ versus 1.38±0.05×10^6^ cell/dish, P<0.001, n = 6, Fig. 4C). Data presented in Fig. 4D show that directional migration of VSMCs stimulated by high glucose were inhibited by palmitic acid (111.11±32.29 versus 32.10±15.42 µm, P<0.001, n = 6, Fig. 4D), and transwell analysis confirmed that the number of migrated cells was fewer in the PA+HG group than in the HG group (37±18.6 versus 107±14.0, P<0.001, n = 6, Fig. 4F).

**Figure 5:**
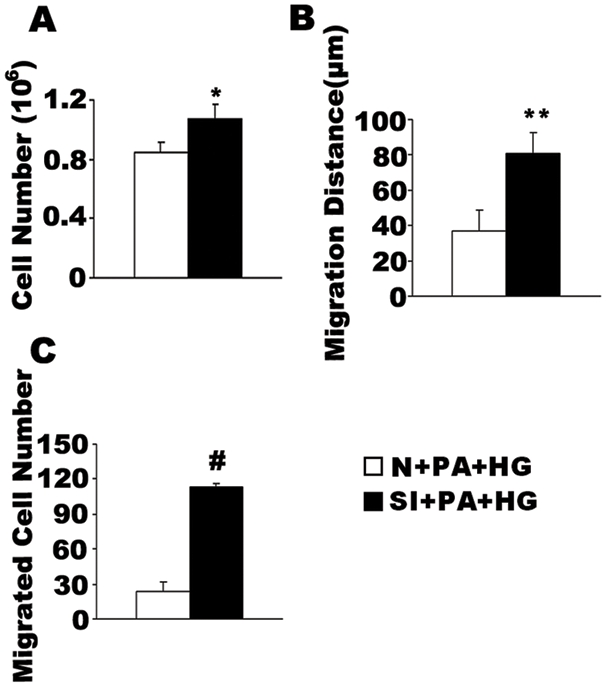
Suppression of PGC-1α abolishes the inhibitory effect of palmitic acid on high glucose-induced VSMC growth and movement. VSMCs were transfected with siRNA (SI) or the negative control (N), then left stimulated with 15 mM gluoose (HG) in the presence of 0.4 mM palmitic acid (PA) for 48 h. Cell growth was determined by counting the number of cells (A). Migration distance was determined by a standard wound healing assay (B) and by transwell analysis (C). The bars represent means±S.E.M (n = 6). *P<0.05, **P<0.01, #P<0.001 compared with the N+PA+HG group.

To further investigate whether the inhibitory effects of palmitic acid on high glucose-induced VSMC proliferation and migration result from its capacity to elevate PGC-1α expression, we transfected VSMCs with siRNA to knock down PGC-1α, and then submitted the cells to the high glucose conditions while in presence of palmitic acid in the medium. We confirmed the interference efficiency and found that PGC-1α expression was decreased by 60–70% at both the mRNA and protein levels (data not shown). Following PGC-1α knockdown, the inhibitory effect of palmitic acid on VSMC proliferation and migration was abolished. As shown in Fig. 5A, in the presence of palmitic acid and high glucose, the number of cells in the siRNA-transfected cells was 1.3-fold higher than the negative control group (1.07±0.10×10^6^ versus 0.85±0.07×10^6^ cell/dish, P<0.05, n = 6, Fig. 5A). In the presence of high glucose and palmitic acid, migration distance was 81±11 µm following PGC-1α knock-down, but 37±12 um in the control group (Fig. 5B), a tendency mirrored in the transwell assay (113±3 versus 23.8±7 cells, P<0.001, n = 6, Fig. 5C). These results indicate that even in the presence of palmitic acid (a strong stimulant of PGC-1α expression), PGC-1α knockdown induces VSMC proliferation and migration, essentially to a level comparable to high glucose alone. Thus, PGC-1α is likely an integration point in VSMCs for nutrient signals, which are likely to engage different signaling pathways upstream of PGC-1α.

### PGC-1α inhibits activation of the ERK1/2 pathway in VSMCs by high glucose

**Figure 6:**
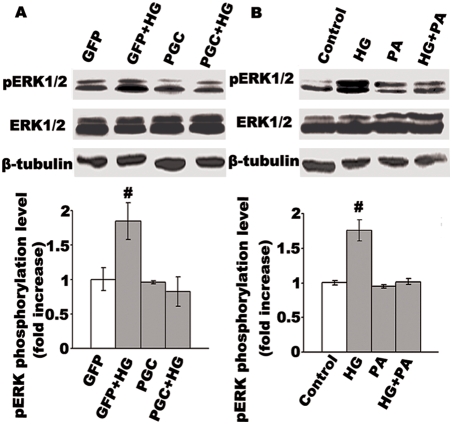
Increase of PGC-1α either exogenously by adenovirus or endogenously by palmitic acid abolished high glucose induced phosphorylation of ERK1/2 in VSMCs. VSMCs were infected with adenovirus for 24 h, then made quiescent by serum-starvation for 6 h, and then stimulated with HG for 4 h. Total protein were harvested and used to detect ERK1/2 activity (A). Cultured VSMCs in 5.5 mM glucose were made quiescent by serum-starvation for 6 h, then cells were either left untreated or stimulated with 15 mM glucose (HG) in the absence and presence of 0.4 mM palmitic acid (PA) for 12 h. ERK activity were determined by Western blotting with ERK1/2 (pT202/pY204) Phospho-Specific antibodies compared with b-tubulin antibody and densitometric analysis of ERK1/2 activity are showed. Representative blots of three similar results are showed. #P<0.001 compared with control.

High glucose stimulates VSMC proliferation and migration through the activation of MAPK ERK signaling [3], [4]. Consistent with these reports, our results show that high glucose activated ERK1/2, a key element of the ERK MAPK pathway. Following PGC-1α overexpression, the ERK1/2 protein in VSMC no longer was activated by high glucose (see Fig. 6A), the phosphorylation of ERK in VSMCs transfected with Ad-PGC-1α decreased approximately 78% compared with that in Ad-GFP infected group under high glucose conditions. Furthermore, palmitic acid, which has been shown to stimulate PGC-1α expression, seemed to have little effect on ERK phosphorylation, but high glucose-induced phosphorylation of ERK almost reverted to the level of control by the addition of palmitic acid (see Fig. 6B). These results suggest that PGC-1α inhibits VSMC proliferation and migration through the inhibition of MAPK ERK pathway.

## Discussion

PGC-1α, an important transcriptional coactivator, plays a key role in energy metabolism [14], [15]. PGC-1α expression is highly related to glucose metabolism, and its expression level changes in various tissues in rodent models of diabetes. It has previously been shown that both mRNA and protein levels of PGC-1α were significantly reduced in adipose tissue from insulin-resistant subjects [23]. Similarly, decreases in the mRNA levels of PGC-1α were found in skeletal muscle from type 2 diabetics [21]. In contrast, PGC-1α mRNA levels are elevated in the liver [17], [20], as well as in hearts of rodents in models of both type 1 and type 2 diabetes [22]. These changes in PGC-1α levels served as a causal factor or as a mediator of the corresponding pathophysiologic processes. In addition to alterations of the insulin/glucagon axis, the direct effect of chronic hyperglycemia may affect the expression level of PGC-1α. In our previous study, we were the first to report that high glucose directly inhibits PGC-1α expression in isolated rat islets [25]. Here, we show that PGC-1α mRNA expression is inhibited in blood vessel media in STZ-diabetic rats. Further experiments revealed that high glucose directly, and dose-dependently, inhibits PGC-1α mRNA expression in cultured rat VSMCs (Fig. 1). These results indicate that high glucose decreases PGC-1α expression in VSMCs both in vivo and in vitro. However, the precise mechanism through which high glucose regulates PGC-1α expression in VSMC needs further study.

A large body of work has established that chronic hyperglycemia promotes VSMC proliferation and migration and contributes to the progress of diabetic atherosclerosis [1]–[3]. Previous studies show that the PPAR family of proteins may be involved in the process [7]. TZD inhibits VSMC proliferation and migration through the activation of PPARγ, which tends to inhibit the expression of several genes involved in ERK-dependent mitogenic response, leading to the inhibition of cell growth and, finally, cell migration [8]–[12]. Given that PGC-1α acts as a transcriptional coactivator for PPARγ, thus regulating many physiological processes [13]–[15], we hypothesized that the downregulation of PGC-1α by glucose may play a role in an in vitro-model of VSMC proliferation and migration induced by hyperglycemia. Our results show that overexpression of PGC-1α by adenoviral infection abolishes (Fig. 2), while suppression of PGC-1α amplifies (Fig. 3) hyperglycemia-induced VSMC proliferation and migration, providing evidence for a direct role of PGC-1α in this process.

As the most prevalent saturated FFA in circulation, palmitic acid has been shown to down-regulate PGC-1α expression in muscle cells [26]. Our previous study found that palmitic acid had no effect on VSMC proliferation and migration, but can markedly increase PGC-1α expression in VSMCs [24]. The mixtures of palmitic acid and high glucose exhibit no stimulatory effect on VSMC proliferation and migration accompanying a persisting induction of PGC-1α (Fig. 4). When PGC-1α is knocked down by siRNA interference, the stimulatory effect of high glucose on VSMC proliferation and migration was restored even in presence of palmitic acid (Fig. 5), indicating that PGC-1α is an integration point downstream of various nutrient-dependent signals that regulate VSMC proliferation and migration.

It was previously shown that high glucose induces mitogenesis in VSMCs through increasing extracellular signal-regulated kinase (ERK) activity [3]–[6]. Activation of ERKs subsequently leads to the phosphorylation and the activation of a number of downstream targets, such as Elk-1 and Ets-1, which evoke c-Fos and MMP-9 and contribute to VSMCs growth and migration, respectively. In the present study, high glucose markedly increased phosphorylated ERK1/2 protein levels in VSMCs, whereas overexpression of PGC-1α completely abolished this phosphorylation (Fig. 6). However, little effect is observed either on VSMC proliferation and migration or on the activity of ERK1/2 when PGC-1α was overexpressed in the VSMCs without high glucose stimulation, indicating that the inhibitory effect of PGC-1α on VSMC proliferation and migration only exists in the cells which have been exposed and responded to the mitogenic signal, instead of quiescent VSMCs. These data suggest PGC-1α inhibits high glucose-induced ERK activity in VSMCs, and negative regulation of PGC-1α in VSMC proliferation and migration is achieved by inhibiting nuclear ERK MAPK signaling. Our results confirmed to the previous report which showed that overexpression of PGC-1α abolished oleic acid induced ERK1/2 activity. However, it also reported that PGC-1α expression was regulated in skeletal muscle cells through a mechanism involving MAPK-ERK and NF-κB activation (26), suggesting the interaction between PGC-1α expression and ERK1/2 phosphorylation are different in various cell types. The differences may attribute to different mechanism involved and may relate to the regulation of specific cell function. In VSMCs, given that PPARγ has been reported to act as an inhibitory factor upstream of the ERK MAPK pathway (9–12) and PGC-1α is a co-activator of PPARγ, the inhibitory effect of PGC-1α on ERK activity may be mediated through the coactivation of PPARγ. The precise mechanisms of PGC-1α regulation on ERK activity merit further study.

In summary, our results suggest that high glucose increases VSMC proliferation and migration through PGC-1α. Experimental elevation of PGC-1α (by adenoviral overexpression or incubation with palmitic acid) inhibits high glucose-induced VSMC proliferation and migration. Thus, our results reveal a novel function of PGC-1α as a regulator of VSMC proliferation and migration, and provide a potential strategy of treatment for diabetic atherosclerosis.

## Materials and Methods

### Ethics Statement

The investigation conforms to the *Guide for the Care and Use of Laboratory Animals* published by the US National Institutes of Health (NIH Publication No. 85-23, revised 1996). All animals were treated in accordance with guidelines established by the Nanjing University Institutional Animal Care and Use Committee.

### Creating a type I diabetes rat model

Sprague-Dawley rats weighing about 180–200 g were made diabetic by a single injection of streptozotocin (55 mg/kg intraperitoneally) according to a previously described method [27], [28]. Blood glucose was monitored for up to 2 weeks, and only the rats with blood glucose >16.6 mM were used for further study. Six weeks after injection of streptozotocin, thoracic arterial samples were obtained, and the intima and outer and inner tissue layers were removed carefully from samples. The vessel mediae was quickly immersed in liquid nitrogen and transferred to a −80°C freezer for later use.

### Cell Culture

Primary vascular smooth muscle cells were isolated from the thoracic aortas of 3- to4-week-old male Sprague-Dawley rats and characterized morphologically and immunohistochemically as described previously [29], [30]. VSMCs were maintained in DMEM (Gibco-Invitrogen, Carlsbad, USA) containing 5.5 mmol/L glucose. VSMCs cultured for 4 to 8 passages were used. In glucose-stimulated experiments, VSMCs were incubated in serum-free DMEM medium containing 5.5, 11, 15 or 25 mmol/L glucose, respectively. In Palmitic acid-stimulated experiments, 0.4 mmol/L Palmitic acid (Sigma) was prepared as described previously [24]. Glucose (1000 mmol/L, dissolved in PBS then sterilized by filtration)were also supplemented to form a final concentration of 15 mmol/L glucose. 15 mM glucose (HG), 0.4 mmol/L Palmitic acid (PA), HG plus PA were then applied to VSMCs for 48 hours before RNA isolation or proliferation and migration assays were performed.

### Adenovirus Infection

Recombinant adenoviruses expressing PGC-1α GFP fusion protein and GFP alone were kindly provided by Dr. Daniel P. Kelly (Center for Cardiovascular Research, Washington University School of Medicine). VSMCs were infected with purified adenovirus at an MOI (multiplicities of infection) of 50 to obtain 95–100% efficiency, as determined by GFP expression 24 h after infection.

### siRNA interference

Three siRNA sequences targeting different sites of rat PGC-1α cDNA were designed and synthesized by Genesil (Wuhan, PR China). Control sequence which could not target PGC-1α cDNA was also included as a negative control. siRNA was transfected into VSMCs using LipofectAmine reagent (Gibco-Invitrogen, Carlsbad, USA) according to the manufacture's manual. Of several sequences tested for PGC-1α knock-down, the sequence with the best interfering effect was selected as described previously [24].

### VSMC Proliferation Assay

Cell counting analysis was performed for evaluation of the effect of glucose on VSMC proliferation. In separate experiments, VSMCs were seeded in 6 cm plastic dishes (1.5×10^5^ cells/dish) and were allowed to grow to subconfluence, then were growth-arrested by serum deprivation for 12 h. Cells were then divided into several groups and were applied to different stimulation. After 48 h, cells were resuspended with 0.05% trypsin and 0.02% EDTA, and the cell number was determined with hemocytometer.

### VSMC Migration Assay

Migration of VSMCs was investigated by a standard *in vitro* wound assay [30], [31], and by the modified Boyden chamber assay [30], [32]. For the woundhealing assay, VSMCs were plated at an initial density of 1×10^5^ cells/well on 6-well plates and were grown to confluence to form a monolayer, then subjected to adenovirus infection or siRNA interference according to the experiment requirements, a plastic cell scraper was then used to make an approximately 2.0 mm–width gap in the cell monolayer, different stimulants were added subsequently (set as 0 time point). A reference point was created on the bottom of the plate in the field of the wound using direct microscopic visualization. The measurement of the width of the gap after 48 h was subtracted from that at 0 h to give the distance the cells migrated. The results of the five readings from each well were averaged. Independent stimulation experiments were repeated three times. Boyden chamber cell migration assay was performed using transwell chambers with fibronection-coated 8-µm-pore-size polycarbonate membranes (BD Biosciences). Preconfluent VSMCs treated in the same way were then suspended in DMEM–0.5% FBS to a concentration of 4×10^5^ cells/mL. Different fatty acids or serum free DMEM (0.6 mL) were added to the lower compartment. A 0.1-mL cell suspension (final concentration, 4×10^4^ cells/well; diameter, 6.5 µm) was added to the upper compartment, and cells were then incubated at 37°C (95% air–5% CO_2_). 6 h later, non-migrated cells were removed with a cotton swab, and the migrated cells were fixed with paraformal-dehyde for 30 min and stained with crystal violet. Cell migration was quantified by blind counting of the migrated cells on the lower surface of the membrane of 5 fields per chamber under microscope.

### RNA Isolation and Analysis

Total RNA was isolated from rat arterial samples or rat VSMCs using RNeasy kit (Qiagen, Hilden, Germany) according to the manufacturer's instructions. cDNA was synthesized and subjected to PCR amplification with the ABI Prism 7000 sequence detection system (Applied Biosystem, Foster City, CA) as described [24]. Sequence-specific primers and Taqman probes for real-time RT-PCR were designed and synthesized by Shinegene Co, Shanghai. The primer sequences used to amplify rat PGC-1α cDNA (GenBank access number AY237127) were 5′-AGGTCCCCAGGCAGTAGAT-3′ (sense) and 5′-CGTGCTCATTGGCTTCATA (antisense), probe: 5′-fam-ATGAATCAAGCCACT ACAGACACC-tamra-3′; primers for rat β-actin (GenBank access number V01217) were 5′-AGGGAAATCGTGCGTGAC-3′ (sense) and 5′-CGCTCATTGCCGATAGT G-3′ (antisense), probe: 5′-fam-CTGTGCTATGTTGCCCTAGACTTC-tamra-3′; Amplification conditions were: one cycle of 95°C for 5 minutes followed by 40 cycles of 95°C for 30 seconds, 60°C for 1 minute, and final one cycle of 72°C for 2 minutes. Relative abundance of mRNA was determined from the C_T_ values and was normalized to the value of the house-keeping gene.

### Western Blot Analysis

For Western blot analysis, total proteins were applied to 7.5% SDS-PAGE gel electrophoresis for PGC-1α detection, and rabbit polyclonal PGC-1 antibody (Santa Cruz Biotechnology, Santa Cruz, USA) was used as primary antibody. Samples containing 50 µg total proteins were applied to 12.5% SDS-PAGE gel electrophoresis for phosphorylated ERK1/2 detection, and phospho-specific mouse anti-human ERK1/2 antibody (BD Biosciences), mouse anti-human ERK antibody (BD Biosciences) and mouse monoclonal GAPDH antibody (Santa Cruz) were used as primary antibody.

### Statistical Analysis

All results are expressed as means±SEM. Data were analyzed using a one-way ANOVA and Student-Newman-Keuls tests for multiple comparisons or Student's test for unpaired data. In all cases P≤0.05 was taken as statistically significant.

## References

[1] Krolewski AS, Warram JH, Valsania P, Martin BC, Laffel LM (1991). Evolving natural history of coronary artery disease in diabetes mellitus.. Am J Med.

[2] Stamler J, Vaccaro O, Neaton JD, Wentworth D (1993). Diabetes, other risk factors, and 12-yr cardiovascular mortality for men screened in the Multiple Risk Factor Intervention Trial.. Diabetes Care.

[3] Suzuki M, Akimoto K, Hattori Y (2002). Glucose upregulates plasminogen activator inhibitor-1 gene expression in vascular smooth muscle cells.. Life Sci.

[4] Igarashi M, Wakasaki H, Takahara N, Ishii H, Jiang ZY (1999). Glucose or diabetes activates p38 mitogen-activated protein kinase via different pathways.. J Clin Invest.

[5] Natarajan R, Scott S, Bai W, Yerneni KK, Nadler J (1999). Angiotensin II signaling in vascular smooth muscle cells under high glucose conditions.. Hypertension.

[6] Hsueh WA, Law RE (1998). Cardiovascular risk continuum: implications of insulin resistance and diabetes.. Am J Med.

[7] Hsueh WA, Law RE (2001). PPARgamma and atherosclerosis: effects on cell growth and movement.. Arterioscler Thromb Vasc Biol.

[8] Law RE, Meehan WP, Xi XP, Graf K, Wuthrich DA (1996). Troglitazone inhibits vascular smooth muscle cell growth and intimal hyperplasia.. J Clin Invest.

[9] Wakino S, Kintscher U, Kim S, Yin F, Hsueh WA (2000). Peroxisome proliferator-activated receptor gamma ligands inhibit retinoblastoma phosphorylation and G1–>S transition in vascular smooth muscle cells.. J Biol Chem.

[10] Goetze S, Xi XP, Kawano H, Gotlibowski T, Fleck E (1999). PPAR gamma-ligands inhibit migration mediated by multiple chemoattractants in vascular smooth muscle cells.. J Cardiovasc Pharmacol.

[11] Graf K, Xi XP, Yang D, Fleck E, Hsueh WA (1997). Mitogen-activated protein kinase activation is involved in platelet-derived growth factor-directed migration by vascular smooth muscle cells.. Hypertension.

[12] Dubey RK, Zhang HY, Reddy SR, Boegehold MA, Kotchen TA (1993). Pioglitazone attenuates hypertension and inhibits growth of renal arteriolar smooth muscle in rats.. Am J Physiol.

[13] Puigserver P, Wu Z, Park CW, Graves R, Wright M (1998). A cold-inducible coactivator of nuclear receptors linked to adaptive thermogenesis.. Cell.

[14] Puigserver P, Spiegelman BM (2003). Peroxisome proliferator-activated receptor-gamma coactivator 1 alpha (PGC-1 alpha): transcriptional coactivator and metabolic regulator.. Endocr Rev.

[15] Lin J, Handschin C, Spiegelman BM (2005). Metabolic control through the PGC-1 family of transcription coactivators.. Cell Metab.

[16] Wu Z, Puigserver P, Andersson U, Zhang C, Adelmant G (1999). Mechanisms controlling mitochondrial biogenesis and respiration through the thermogenic coactivator PGC-1.. Cell.

[17] Yoon JC, Puigserver P, Chen G, Donovan J, Wu Z (2001). Control of hepatic gluconeogenesis through the transcriptional coactivator PGC-1.. Nature.

[18] Lin J, Wu H, Tarr PT, Zhang CY, Wu Z (2002). Transcriptional co-activator PGC-1 alpha drives the formation of slow-twitch muscle fibres.. Nature.

[19] St-Pierre J, Drori S, Uldry M, Silvaggi JM, Rhee J (2006). Suppression of reactive oxygen species and neurodegeneration by the PGC-1 transcriptional coactivators.. Cell.

[20] Herzig S, Long F, Jhala US, Hedrick S, Quinn R (2001). CREB regulates hepatic gluconeogenesis through the coactivator PGC-1.. Nature.

[21] Patti ME, Butte AJ, Crunkhorn S, Cusi K, Berria R (2003). Coordinated reduction of genes of oxidative metabolism in humans with insulin resistance and diabetes: Potential role of PGC1 and NRF1.. Proc Natl Acad Sci U S A.

[22] Finck BN, Lehman JJ, Leone TC, Welch MJ, Bennett MJ (2002). The cardiac phenotype induced by PPARalpha overexpression mimics that caused by diabetes mellitus.. J Clin Invest.

[23] Hammarstedt A, Jansson PA, Wesslau C, Yang X, Smith U (2003). Reduced expression of PGC-1 and insulin-signaling molecules in adipose tissue is associated with insulin resistance.. Biochem Biophys Res Commun.

[24] Zhang Y, Liu C, Zhu L, Jiang X, Chen X (2007). PGC-1alpha Inhibits Oleic Acid Induced Proliferation and Migration of Rat Vascular Smooth Muscle Cells.. PLoS ONE.

[25] Zhang P, Liu C, Zhang C, Zhang Y, Shen P (2005). Free fatty acids increase PGC-1alpha expression in isolated rat islets.. FEBS Lett.

[26] Coll T, Jove M, Rodriguez-Calvo R, Eyre E, Palomer X (2006). Palmitate-mediated downregulation of peroxisome proliferator-activated receptor-gamma coactivator 1alpha in skeletal muscle cells involves MEK1/2 and nuclear factor-kappaB activation.. Diabetes.

[27] Zhao W, Devamanoharan PS, Henein M, Ali AH, Varma SD (2000). Diabetes-induced biochemical changes in rat lens: attenuation of cataractogenesis by pyruvate.. Diabetes Obes Metab.

[28] Ramana KV, Friedrich B, Srivastava S, Bhatnagar A, Srivastava SK (2004). Activation of nuclear factor-kappaB by hyperglycemia in vascular smooth muscle cells is regulated by aldose reductase.. Diabetes.

[29] Fujiwara R, Hayashi T, Nakai T, Miyabo S (1994). Diltiazem inhibits DNA synthesis and Ca2+ uptake induced by insulin, IGF-I, and PDGF in vascular smooth muscle cells.. Cardiovasc Drugs Ther.

[30] Majack RA, Clowes AW (1984). Inhibition of vascular smooth muscle cell migration by heparin-like glycosaminoglycans.. J Cell Physiol.

[31] Sarkar R, Meinberg EG, Stanley JC, Gordon D, Webb RC (1996). Nitric oxide reversibly inhibits the migration of cultured vascular smooth muscle cells.. Circ Res.

[32] Grotendorst GR, Seppa HE, Kleinman HK, Martin GR (1981). Attachment of smooth muscle cells to collagen and their migration toward platelet-derived growth factor.. Proc Natl Acad Sci U S A.

